# Editorial: Plant immune responses, evolutionary dynamics, control and transmission of plant ssDNA viruses, volume II

**DOI:** 10.3389/fpls.2025.1768424

**Published:** 2026-01-19

**Authors:** José T. Ascencio-Ibáñez, Rafael F. Rivera-Bustamante, Björn Krenz

**Affiliations:** 1 Department of Molecular and Structural Biochemistry, North Carolina State University, Raleigh, NC, United States; 2 Centro de Investigación y Estudios Avanzados (CINVESTAV), Unidad Mérida, Mérida, Yuc, Mexico; 3 Leibniz Institute Deutsche Sammlung von Mikroorganismen und Zellkulturen GmbH-German Collection of Microorganisms and Cell Cultures, Braunschweig, Germany

**Keywords:** antiviral immunity, geminivirus, miRNA - microRNA, nanovirus, SEGS

Plant ssDNA viruses, particularly members of the families *Geminiviridae* and *Nanoviridae*, continue to pose major threats to global food security. Their capacity to rapidly evolve, circumvent host immunity, and exploit diverse transmission routes demands sustained scientific attention. Building on the momentum generated by the first volume of this Research Topic, *Plant Immune Responses, Evolutionary Dynamics, Control and Transmission of Plant ssDNA Viruses: Volume II* brings together five studies that expand our mechanistic, ecological, and applied understanding of plant–virus interactions. Collectively, these contributions highlight not only the sophistication of ssDNA viruses but also the multifaceted layers of host immunity that shape viral success or failure.

The study by Rajabu et al. provides key insights into the biological activity of SEGS-1, an endogenous DNA sequence previously identified in cassava. By introducing SEGS-1 into Arabidopsis, the authors reveal that it enhances symptom severity and viral accumulation even outside its native host, and notably, without forming episomal DNAs. This demonstrates that SEGS-1 functions as a *genomic enhancer* of viral infection and not solely through episome formation, as previously proposed. Their work underscores an important conceptual advance: plant genomes may harbor latent sequences capable of modulating ssDNA virus infection, potentially acting as co-factors during disease outbreaks. These findings broaden the framework for understanding how viral pathogenesis can be shaped by host-encoded elements that inadvertently potentiate virus replication *in trans*. Similarly, Chiunga et al. investigate the enigmatic SEGS-1 element from another angle, asking how its episomal forms arise in cassava and how they influence cassava mosaic disease (CMD). Their results reveal striking genotype-dependent differences: while some cassava varieties readily generate SEGS-1 episomes during CMB infection, others do not. Moreover, episome-forming varieties exhibit stronger CMD symptoms, particularly those carrying CMD2-type resistance, which SEGS-1 can partially overcome. These findings illuminate a previously underappreciated layer of CMD epidemiology: the capacity of a host genotype to produce episomal SEGS-1 may act as a virulence-enhancing factor, shaping disease severity and resistance durability. The study highlights the importance of integrating host-specific genomic responses into breeding strategies aimed at long-term resistance.

Two other studies deepen our understanding of ssDNA virus transmission dynamics and epidemiology. Mendoza et al. present a striking new discovery: *Canna indica*, a widely cultivated ornamental plant, not only supports *Banana bunchy top virus* (BBTV) infection but can also transmit the virus via seeds. This is the first evidence of seed transmission for a nanovirus and has profound implications for disease spread, given the popularity of ornamental plant trade and the generally unregulated movement of non-crop species. The authors demonstrate that BBTV accumulates in both embryos and endosperm and that seedlings derived from infected seeds are fully competent to transmit the virus through vector aphids. On the defensive side of the plant–virus arms race, Sampaio et al. provide a comprehensive review of the LRR-RLK Subfamily II signaling modules and their functions in antiviral immunity. They bring clarity on how co-receptors such as NIKs, SERKs, and CIKs act as crucial nodes linking developmental pathways, PTI, and antiviral responses. The authors summarize that NIK1-mediated translational suppression operates as a conserved antiviral mechanism and emphasize how viruses, especially begomoviruses, have evolved dedicated suppressors, such as NSP, to counteract this defense. This review highlights LRRII-RLKs as promising targets for genetic manipulation: engineered activation of NIK1, for instance, has already demonstrated strong broad-spectrum virus resistance.

By integrating mechanistic studies across model and crop species, the article provides a conceptual roadmap for leveraging co-receptor signaling to enhance antiviral immunity in the field. Finally, Pandey et al. employ an *in-silico* pipeline to identify chili-derived microRNAs predicted to target a broad set of begomovirus genes. While computational, their work provides a valuable foundation for designing artificial miRNAs and gene-silencing strategies aimed at suppressing essential viral functions. By converging predictions from five independent algorithms, they identify five strong candidate miRNAs with robust complementarity to chili leaf curl virus genes including Rep, TrAP, REn, and βC1. The study can accelerate the early stages of antiviral resistance development and underscores the RNA-based strategies for controlling ssDNA viruses.

Together, these five contributions highlight important applied implications for breeding, diagnostics, and disease management in key crops including cassava, banana, and chili peppers. We thank all authors and reviewers for their contributions and hope this Research Topic stimulates further interdisciplinary research aimed at improving plant health and securing agricultural productivity in the face of evolving viral threats.

